# The Efficacy of Dietary Interventions in Patients with Gastroesophageal Reflux Disease: A Systematic Review and Meta-Analysis of Intervention Studies

**DOI:** 10.3390/nu16030464

**Published:** 2024-02-05

**Authors:** Narisorn Lakananurak, Panyavee Pitisuttithum, Paweena Susantitaphong, Tanisa Patcharatrakul, Sutep Gonlachanvit

**Affiliations:** 1 Division of Clinical Nutrition, Department of Medicine, Faculty of Medicine, King Chulalongkorn Memorial Hospital, Chulalongkorn University, The Thai Red Cross Society, Bangkok 10330, Thailand; narisorn.l@chula.ac.th; 2 Division of General Internal Medicine, Department of Medicine, Faculty of Medicine, King Chulalongkorn Memorial Hospital, Chulalongkorn University, The Thai Red Cross Society, Bangkok 10330, Thailand; 3 Center of Excellence in Neurogastroenterology and Motility, Faculty of Medicine, Chulalongkorn University, Bangkok 10330, Thailand; dr_tanisa@yahoo.com (T.P.); sutep.g@chula.ac.th (S.G.); 4 Division of Nephrology, Department of Medicine, Faculty of Medicine, King Chulalongkorn Memorial Hospital, Chulalongkorn University, The Thai Red Cross Society, Bangkok 10330, Thailand; paweena.s@chula.ac.th; 5 Center of Excellence for Metabolic Bone Disease in CKD Patients, Faculty of Medicine, Chulalongkorn University, Bangkok 10330, Thailand; 6 Division of Gastroenterology, Department of Medicine, Faculty of Medicine, King Chulalongkorn Memorial Hospital, Chulalongkorn University, The Thai Red Cross Society, Bangkok 10330, Thailand

**Keywords:** diet, food, dietary therapy, gastroesophageal reflux, GERD, meta-analysis

## Abstract

Background: International guidelines recommend dietary interventions as one of the most important treatments for patients with gastroesophageal reflux disease (GERD). Evidence to confirm the efficacy of these treatment modalities is lacking. The present study aims to evaluate the efficacy of dietary interventions on GERD-related outcomes evaluated in intervention studies on GERD patients. Methods: A systematic review and meta-analysis was performed according to PRISMA. The PubMed/MEDLINE, Web of Sciences, and Scopus databases were utilized for the literature search. Two independent researchers searched for relevant publications published up until June 2023. Intervention studies evaluating the efficacy of dietary interventions in patients with GERD were included. Results: A total of 577 articles were identified during the initial literature search. After reviewing, 21 studies with 16 different types of dietary interventions were included in the analysis. The interventions were divided into low-carbohydrate diets (3 studies), high-fat diets (2 studies), speed of eating studies (3 studies), low-FODMAP diets (2 studies), and other interventions (12 studies). A meta-analysis could be performed for low-carbohydrate diets and speed of eating interventions. Low-carbohydrate diets resulted in a significant reduction in esophageal acid exposure time (mean difference = −2.834%, 95% confidence interval (CI): −4.554 to −1.114), while a slow speed of eating did not lead to a lower percentage of reflux events compared to fast eating (risk ratio = 1.044, 95% CI: 0.543–2.004). Most other interventions showed positive effects in only a single study. Conclusion: Low-carbohydrate diets showed a significant improvement in GERD-related outcomes, while a slow eating speed did not result in a reduction in reflux events. The overall evidence regarding dietary interventions in GERD remains scarce. High-quality, long-term RCTs are still required to confirm the effects of dietary interventions in GERD patients.

## 1. Introduction

Gastroesophageal reflux disease (GERD) is a common disease worldwide. According to population-based studies, pooled prevalence of GERD, defined by at least weekly GERD symptoms, was 13%, with a prevalence of more than 25% in some geographic regions [1,2,3]. Diet has been theorized to be associated with the aggravation of GERD symptoms. Avoidance of trigger diets is one of the main treatment modalities and is recommended in current guidelines [4]. These recommendations, however, are largely based on uncontrolled studies. Most of these studies focus on diet as a risk factor of GERD, but do not focus on dietary interventions and their effect on improvement of GERD-related outcomes [5,6].

Studies assessing the benefits of various dietary interventions and GERD-related outcomes have been published recently. The efficacy of dietary interventions such as low-fat, the type/amount of carbohydrates, and low-Fermentable Oligo-, Di-, Mono-saccharides and Polyol (FODMAP) diets have been mostly evaluated in observational studies or pathophysiology-proven studies. For example, a cross-sectional study revealed that a high-fat diet was associated with worsening GERD symptoms [7]. A diet with a high-FODMAP content was found to increase transient, lower esophageal sphincter relaxations (TLESRs), which is the main mechanism of GERD and overlaps with non-constipated irritable bowel syndrome (IBS) [8].

A systematic review by Zhang et al., in 2021, focusing on the correlation between diet and GERD, excluded randomized control studies with dietary interventions and therefore could not elucidate the true effect of diet on GERD [9]. A recent systematic review and meta-analysis by Martin et al., in 2022, included studies with various dietary interventions and evaluated patients with GERD and functional dyspepsia (FD). Based on Rome IV criteria, GERD and FD are different disease entities with their own diagnostic criteria and pathophysiology. The inclusion of both disorders might influence clinical implications, especially in patients without overlapping diseases [10].

At present, even though dietary interventions are generally recommended in clinical practice, a systematic review and meta-analysis focusing on the effect of dietary interventions specifically in patients with GERD is lacking. We conducted a systematic review and meta-analysis of dietary interventions in adults with GERD in order to evaluate the effectiveness of dietary treatments on GERD-related outcomes.

## 2. Materials and Methods

### 2.1. Search Strategy

Two independent researchers searched PubMed/MEDLINE, Web of Sciences, and Scopus for relevant publications up until June 2023. The systematic search was conducted using Medical Subject Heading (MeSH) together with non-MeSH keywords for titles and abstracts including: “Diet” OR “Food” OR “Dietary Pattern” OR “Food Pattern” AND “Gastroesophageal Reflux” OR “GERD” OR “Gastric acid reflux” OR “Gastroesophageal reflux disease” OR “Esophageal reflux” OR “Heart burn” OR “Barrett’s esophagus” OR “Reflux esophagitis”. No restrictions on the language, time of publication, and study location were applied. Duplication of the studies was further detected using Covidence.

### 2.2. Inclusion and Exclusion Criteria

Study eligibility was defined according to the Participant, Intervention, Comparator, Outcome, Study type (PICOS) framework [11]. Inclusion criteria for this study included studies that were performed on adult patients (more than 18 years of age) with a diagnosis of GERD. GERD was defined according to the American College of Gastroenterology as the condition in which reflux of gastric contents into the esophagus results in symptoms and/or complications [4]. Only intervention studies evaluating all components of the diet were included. All studies were independently evaluated by two independent researchers. Discrepancies between the researchers were resolved through discussion.

**Figure 1:**
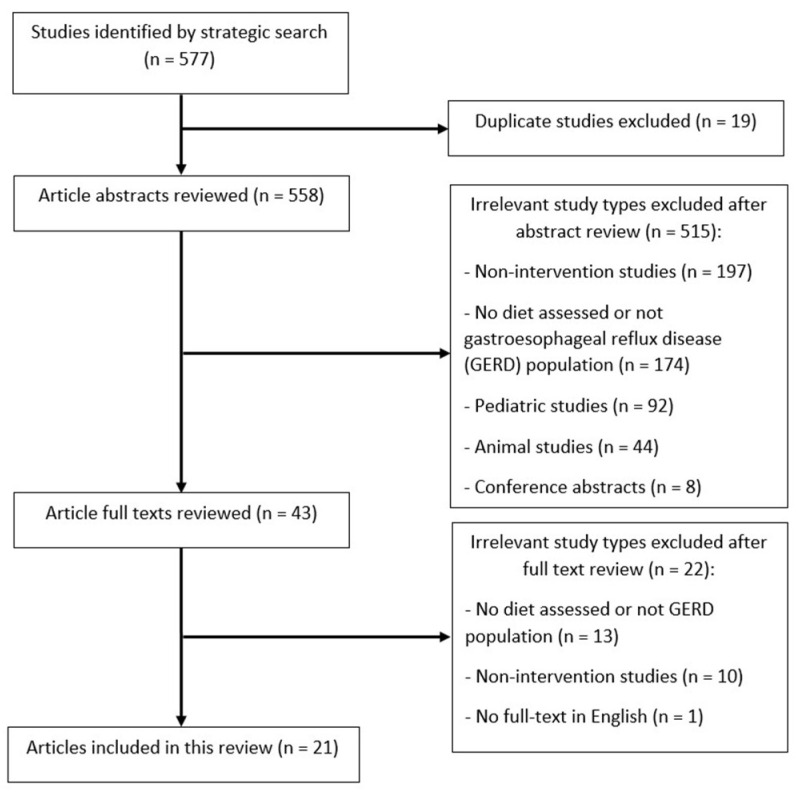
Flow diagram of systematic review.

A total of 577 articles were identified during the initial search and 19 duplicated articles were removed. The remaining studies were screened based on their title and abstract, and 515 irrelevant studies were excluded. The remaining 43 studies were reviewed in greater detail. After full-text reviewing, 22 studies were excluded due to irrelevance, mainly because the studies either lacked a dietary assessment, did not specify only a GERD diagnosis, or were non-intervention studies. In total, 21 articles were included in this study (Figure 1).

### 2.3. Quality Assessment

The quality of the randomized controlled studies in this review was evaluated using the Jadad scale. The scoring system has a total score of 5, evaluating randomization (2 points), blinding (2 points), and withdrawal (1 point). A total score of ≤3 was categorized as low quality. The Newcastle–Ottawa scale was utilized to assess the quality of non-randomized control studies. A maximum score of 9 comprises study group selection (4 points), comparability (2 points), and outcomes (3 points). A total score of ≤3 was considered to indicate low quality; 4–6, medium quality; and ≥7, high quality.

Two reviewers evaluated the quality of each study independently. The results were compared and discussed between the reviewers to reach consensus on any disparities. Major disagreements were brought to a third reviewer to reach a consensus.

### 2.4. Data Extraction and Synthesis

Data extraction was performed by two independent researchers, utilizing the Covidence program. Any disagreement was discussed and resolved accordingly. For each article, the name of the study, the first author’s name, the publication year, study location, study period, study design, sample size, study population demographics (e.g., age, sex, body mass index (BMI)), dietary intervention and control, and outcomes (all reported data on associations between GERD and diet) were extracted.

All findings were narratively synthesized. Meta-analysis was also performed using the Comprehensive meta-analysis software (version 2) when two or more studies had sufficient clinical homogeneity in their intervention and comparative characteristics. Continuous data were reported using mean change. Binary data were assessed and reported using a risk ratio (RR). Heterogeneity was evaluated with the I^2^ statistic, where a value > 50% was considered to represent substantial statistical heterogeneity. A *p*-value of less than 0.05 was considered statistically significant.

## 3. Results

### 3.1. Study Characteristics

The 21 studies included in this review were published between 1998 and 2022. Sixteen different types of dietary interventions in GERD patients were evaluated. Each study evaluated one type of dietary intervention except for one study by Fan et al., which assessed two types of dietary interventions (a high-fat diet and functional food) [12]. We grouped the dietary interventions into low-carbohydrate diets (3 studies), high-fat diets (2 studies), speed of eating studies (3 studies), low-FODMAP diets (2 studies), and other interventions (12 studies). The majority of studies were RCTs (15 studies), of which 8 had cross-over designs.

The greatest number of studies were conducted in the USA (five studies), followed by Turkey (three studies), Italy (two studies), and other countries (Taiwan, China, France, Thailand, Sweden, Singapore, Australia, Mexico, Brazil, Russia, and Iran; each one study). The number of study participants ranged from 8 to 351. Women were predominant in 16 out of 21 studies (76.2%). BMI was documented in 13 studies, revealing that 38% of these studies were conducted on obese individuals (with a BMI ≥ 30 kg/m², or ≥25 kg/m² in Asian populations) [13].

The diagnosis of GERD was based on symptoms in 17 studies, followed by symptoms and endoscopy (2 studies), symptoms and pH monitoring (1 study), and endoscopy and pH monitoring (1 study). The outcomes regarding GERD symptoms were most frequently evaluated, in 17/21 studies (81%). Outcomes associated with pH measurement and quality of life (QoL) were assessed in 13 and 2 studies, respectively. The duration of interventions ranged from immediately post one-meal ingestion to 9 weeks (one meal in 7/21 studies, 33%). All outcomes were measured at the end of the dietary interventions without long-term follow-up.

A review of the quality of the randomized controlled studies (n = 15) revealed that most studies had a low quality on the Jadad scale (11 studies). In addition, the quality of the non-RCT studies (n = 6) was rated as low in five studies and medium in one study (Table 1).

### 3.2. Outcomes of the Studies

Overall, 14 dietary interventions demonstrated significant effects in GERD patients, while non-statistically significant outcomes were found in 2 interventions. The details of the effects of each dietary intervention, categorized by type of diet, are described as follows and in Table 2.

### 3.3. Low-Carbohydrate Diets

Three studies were identified that demonstrated a reduction in GERD symptoms after low-carbohydrate diets [14,15,16]. However, meta-analysis could not be performed due to the difference in their GERD-related symptom measurements. A 2006 study on GERD patients with obesity (BMI > 30 kg/m^2^) (n = 8) conducted by Ausin et al. showed that a low-carbohydrate diet (less than 20 g/day) for 3–6 days could significantly reduce GERD symptoms as evalauted by the GERD Symptom Assessment Scale-Distress Subscale (GSAS-ds) [14]. A RCT cross-over study by Wu et al. evaluating the effects of high- and low-carbohydrate liquid diets (n = 12) found more heartburn and acid regurgitation in the high-carbohydrate liquid diet group [15]. A recent randomized controlled study by Gu et al. evaluating the effect of the amount and types of carbohydrate in GERD patients with obesity divided participants into four groups: high total/high simple carbohydrate (HTHS) (control group), high total/low simple carbohydrate (HTLS), low total/high simple carbohydrate (LTHS), and low total/low simple carbohydrate (LTLS) diets. They found that there was a significant reduction in the total GERD-Q scores in the HTLS, LTHS, and LTLS groups [16].

In terms of pH monitoring measurement, all three studies showed an improvement in their pH monitoring parameters. Austin et al.’s study showed a significant reduction in the 24 h esophageal acid exposure time (AET) (5.1 ± 1.3% before diet vs. 2.5 ± 0.6% post diet; *p* = 0.022). [14] Wu et al. found that Johnson–DeMeester scores, the number of reflux periods, total reflux time, and number of reflux periods longer than 5 min were higher in high-carbohydrate diet (*p* < 0.05). [15] Gu et al. found that both the HTLS and LTHS diet groups had a significant reduction in their 24 h AET, total number of reflux episodes, and number of reflux episodes longer than 5 min compared to baseline. [16]

**Figure 2:**
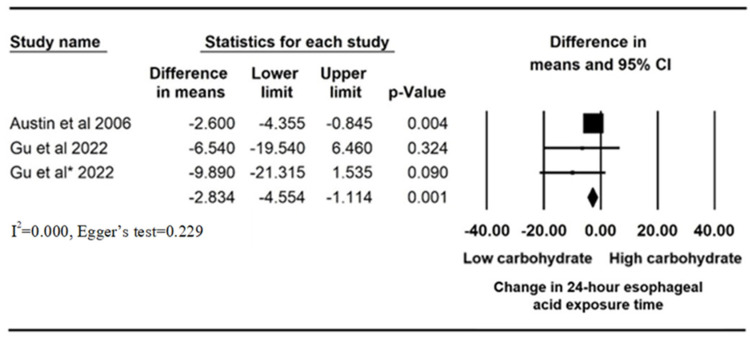
Mean difference of 24 h esophageal acid exposure time (%)pre- and post low-carbohydrate diets. Austin et al., 2006 [14]; Gu et al., 2022 [16]: low total/high simple carbohydrate (LTHS) diet; Gu et al., 2022 * [16]: low total/low simple carbohydrate (LTLS) diet.

**Figure 3:**
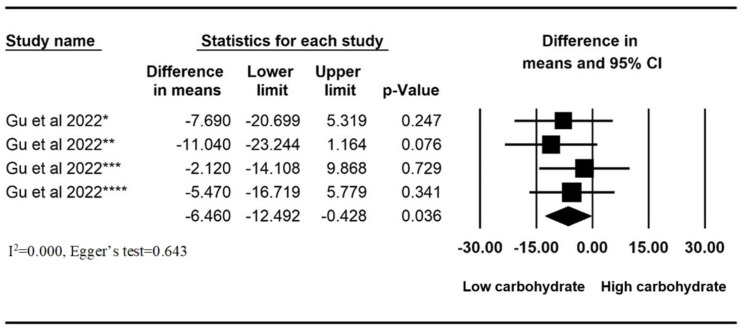
Mean difference of pre- and post−24 h esophageal acid exposure time (%) compared between high- and low-carbohydrate diets among each study arms in a study by Gu et al., 2022 [16] Gu et al., 2022 * [16]: high total/high simple carbohydrate (HTHS) vs. low total/high simple carbohydrate (LTHS) diet; Gu et al., 2022 ** [16]: high total/high simple carbohydrate (HTHS) vs. low total/low simple carbohydrate (LTLS) diet; Gu et al., 2022 *** [16]: high total/low simple carbohydrate (HTLS) vs. LTHS; Gu et al., 2022 **** [16]: high total/low simple carbohydrate (HTLS) vs. LTLS.

Meta-analysis was performed to measure the mean difference of 24 h AET (%)pre- and post low-carbohydrate diets in two studies. A significant reduction in AET was found after the ingestion of low-carbohydrate diets (mean difference = −2.834%, 95% confident interval (CI): −4.554 to −1.114, *p* = 0.001, I^2^ = 0.000, Egger’s test = 0.229) (Figure 2). We also performed a meta-analysis of the study data by Gu et al. to compare the mean difference of the pre- and post AETs of the high- and low-carbohydrate diets in each study arm. Low-carbohydrate diets resulted in a significant reduction in AET compared to high-carbohydrate diets (mean difference = −6.460%, 95% CI: −12.492 to −0.428, *p* = 0.036, I^2^ = 0.000, Egger’s test = 0.643) (Figure 3).

### 3.4. High-Fat Diets

Two randomized cross-over studies evaluated the effect of diets with different fat content in GERD patients. A 1998 study by Penagini et al. compared a high-fat meal (44 g fat, carbohydrate:fat:protein (C:F:P) 39:52:9%) and a balanced meal (20 g fat, C:F:P 60:24:16%). There was no significant difference in the esophageal acid exposure and rate of reflux episodes (number per hour) within 3 h between the two groups [17]. In contrast, another study in 2018, comprising 27 patients with GERD (12 non-erosive reflux disease (NERD) and 15 reflux esophagitis (RE)), revealed a significantly higher percentage of esophageal acid exposure at 4 h (median 5.2% vs. 4%) in the RE group when comparing a high-fat meal (53.7 g fat, C:F:P 29.1:60.6:9.3%) to a standard meal (22.2 g fat, C:F:P 12.3:25:62.6%). However, there was no significant difference in the number of postprandial reflux symptoms between the two groups [12].

### 3.5. Low-FODMAP Diets

A RCT cross-over study by Plaidum et al. compared the acute effects of rice (low FODMAP) and wheat noodle meals (high FODMAP) (n = 8), and found lower regurgitation symptom severity 2 h after lunch with the rice meal. [8] Another study in proton pump inhibitor-refractory GERD patients showed a non-significant improvement in outcomes between low-FODMAP and usual dietary groups [8].

### 3.6. Eating Speed

Three randomized cross-over studies studying eating speed were identified. Fast (within 5 min) and slow (within 30 min) eating were compared in 46 patients with GERD and no statistical significant difference in total reflux events was revealed within 3 h of ingestion [18]. Another study in 60 patients with GERD also showed no statistical difference in terms of the total reflux events, total reflux time, and reflux symptoms within 3 h among patients with normal or abnormal pH monitoring when comparing fast (within 5–10 min, mean 8.4 min) and slow eating protocols (within 25–30 min, mean 27.7 min) [19]. Additionally, no significant difference in the total reflux events and time was found in a recent study of 26 GERD patients with obesity (BMI > 30 kg/m^2^) [20]. We also analyzed the number of reflux events per patient from these three studies and found no significant difference between fast and slow eating speeds (risk ratio = 1.044, 95% CI: 0.543–2.004, *p* = 0.898, I^2^ = 0.000, Egger’s test = 0.861).

### 3.7. Other Dietary Interventions

We found an additional 11 studies showing the positive effects of other types of dietary interventions in GERD patients. A single-blinded RCT study comparing dietary supplements (melatonin, vitamins, and amino acids) and a daily regimen of 20 mg omeprazole showed a significant reduction in GERD symptoms in the dietary supplement group (100% in the dietary supplement group vs. 65.7% in the omeprazole group, *p* = 0.001). The efficacy of the treatment in this study, however, was the time taken (in days) for the patient to achieve their first 24 h without GERD symptoms and 90% of patients reported somnolence in the dietary supplement group [21]. The effect of eating a curry meal on GERD was evaluated in 25 NERD patients post 400 mL and 800 mL of curry ingestion, resulting in a significant increase in the amount of time taken to reach pH < 4 at 4 h from 5.8 ± 1.4 to 15.3 ± 3.1 (*p* < 0.001). Curry also significantly worsened reflux symptoms from 15 to 150 min after ingestion [23]. An RCT pilot study showed that alow vera syrup alleviated heartburn symptoms, but the effect was smaller than that of omeprazole and ranitidine [24]. A randomized cross-over study in 12 NERD patients evaluating the effect of functional foods (marine collagen peptides, wheat oligopeptides, vegetable fat powder, glucose-maltodextrin, isomaltooligosaccharide, extracts of Amomum villosum, tangerine peel, and jujube, composite minerals, vitamins, and other minor ingredients) revealed a lower number of postprandial reflux symptoms compared to a standard meal (median 0 vs. 3 events) [12].

A prospective study reported that soluble dietary fiber ingested for 10 days in NERD patients with a low fiber intake (less than 20 g/d) had a significant benefit, and achieved a 7-day heartburn-free period in 60% of patients and a reduction in their GERD-Q scores. However, this study failed to demonstrate a reduction of in 24 h pH to below 4 after the intervention [25]. A randomized controlled study demonstrated that fermented soy supplementation improved QoL, but only in terms of some indicators [26]. Another RCT found that 3 g of prebiotic whole-plant sugar cane flour (PSCF) daily lead to improvements in heartburn scores (−2.2; 95% CI: −4.2 to −0.14; *p* = 0.037) and total symptom scores (−3.7; 95% CI: −7.2 to −0.11; *p* = 0.044) in 40 GERD patients [27]. A prospective study comparing a liberal diet and a restrictive diet showed that, after instructions on a restrictive diet and reading literature about good and bad food and the provision of a list of good menus for 2 days, subjects showed a significant reduction of AET measured by 48 h pH monitoring among participants with abnormal AET. No symptom change, however, was found after 2 days of dietary adjustment [28].

A prospective study in 3-month proton pump inhibitor (PPI)-unresponsive GERD patients showed a significant reduction in GERD symptoms after a low-nickel diet for 8 weeks [29]. An RCT study evaluating the effect of a diet containing non-caloric sweeteners revealed a significant improvement of burning and retrosternal pain in the non-caloric-sweetener-free group (15% of participants in pre-treatment compared to 0% of participants post treatment, *p* = 0.02) [30]. A recent RCT cross-over study showed that dewaxed coffee (DC) was associated with an increase in heartburn-free days (%) compared to standard coffee (79.82 + 10.84% vs. 50.18 + 17.46%, *p* < 0.05) and improved the quality of life of participants, as measured by the Patient Assessment of Upper Gastrointestinal Disorders-Quality of Life [31]. In contrast, one dietary intervention failed to show significant effects in GERD patients. Dietary nitrate had no significant effect on TLESR, reflux episodes, gastric pH, or reflux symptoms [32].

## 4. Discussion

Dietary intervention is an important and commonly recommended treatment modality for GERD patients. Its evidence, however, has been largely based on observational and epidemiological studies. Intervention studies are crucial to confirm the true efficacy of these interventions.

In the present study, low-carbohydrate diets were found to be the most consistent dietary intervention that showed positive effects on GERD-related outcomes, including symptoms and pH measurements. Our meta-analysis also showed a significant reduction in esophageal acid exposure in low-carbohydrate diets compared to high-carbohydrate diets. The mechanisms underlying the effectiveness of low-carbohydrate diets are not fully understood. It is thought to be related to reduced gastric distension. As for the lower caloric density of carbohydrates, an isocaloric diet with a higher carbohydrate content would occupy a greater gastric volume than one with higher calories from fat [14]. Additionally, the ingestion of some types of carbohydrates, such as lactose and FODMAP, resulted in an increased number of TLESRs in previous physiological studies [22,33]. Interestingly, the effect of low-carbohydrate diets was not related to weight loss, since the benefits could be found even in short-term studies without significant weight reduction seen [16].

While incorporating a slow eating speed is often advised in clinical practice, different eating speeds consistently showed no effect on GERD-related outcomes in all three identified intervention studies and our meta-analysis. However, these studies were all conducted by the same study group in Turkey. In addition, the quality of these studies was low. These factors limit these studies’ generalizability and are a cause for further studies to test slow eating interventions.

We found inconsistent effects of low-fat and low-FODMAP diets on GERD-related outcomes in our systematic review. Positive effects on outcomes were found in one study, but not in a second study, with both interventions. Physicians often advise patients to avoid eating diets with a high-fat content, as it could delay gastric emptying and increase the reflux of gastric content [34,35]. Consistent results supporting this recommendation were not evidenced in previous intervention studies. The level of fat content may be an important factor and may explain this inconsistent result. A very high fat content seems to be a prerequisite for GERD, as the study using the highest proportion of fat (60%) [12] resulted in significantly higher reflux compared to a lower fat content (50%) [17]. With regard to FODMAP, the positive study evaluated only one type of FODMAP (wheat) [8], compared to the negative study that advised patients to restrict all food with a high FODMAP content [36]. The inconsistent outcomes found in these studies may be due to other substances found in wheat rather than FODMAPs (e.g., gluten). A previous study found that gluten-free diets relieved GERD-related symptoms in a significantly higher proportion of celiac disease patients with NERD than non-celiac disease patients (86.2% vs. 66.7%) [37]. However, this study mainly focused patients with celiac disease and, therefore, it was not included in our study. This inconsistency in results may also be due to different study populations. The negative study included patients with refractory GERD, which, in reality, were more accurately classified as non-GERD and functional gastrointestinal disorders [38,39,40].

Most of the other dietary interventions showed significant effects on some GERD-related outcomes, except for the dietary nitrate intervention. However, the effect of each dietary intervention was elucidated in only one study. The positive results of most published studies may be due to publication bias, since studies with positive outcomes are more commonly accepted for publication [41]. For this reason, more studies are needed to confirm the positive effects of these dietary interventions.

Two previous systemic reviews showed an association between multiple dietary factors and GERD [9,42]. Most of these factors have never been tested in intervention studies, including citrus fruits, carbonated beverages, spicy fried food, skipping breakfast, eating very hot food, vegetarian diets, and meat restriction [42]. High-fat diets and fast eating were associated with GERD in these studies ((OR) of 7.568, 95% CI: 4.557–8.908, and OR of 4.06, 95%: CI 3.11–5.29, respectively) [9]. These findings, however, were based on cross-sectional and case–control studies.

Only one recent systematic review and meta-analysis specifically focused on intervention studies regarding dietary interventions and GERD [10]. Only two studies evaluating ginger supplements could be identified for inclusion in that meta-analysis [43,44]. These studies showed a significant improvement of GERD symptoms (OR 7.50 (95% CI: 3.62–15.54)). However, the two studies included patients with either GERD or FD, with a primary focus on patients with FD. Though GERD and FD commonly overlap, they are different diseases and contain different pathophysiological etiologies [45,46]. Two studies on low-carbohydrate diets and one study on a low-FODMAP diet were included in the previous study, and these have also been included in our study. One study on a low-fat diet, included in the previous study, was excluded in our study because patients with FD were also included [47].

To the best of our knowledge from a review of the literature, this is the first systematic review and meta-analysis focusing on dietary interventions specifically tested in patients with GERD. The findings of our meta-analysis can be utilized for guidance in terms of dietary advice from clinicians who treat this common gastrointestinal problem. Moreover, the effect of low-carbohydrate diets and eating speed was firstly elucidated in our meta-analysis.

This study is not without some important limitations. Firstly, the intervention studies mostly contained small numbers of participants within a heterogeneous population. They were also generally conducted over various lengths of time, and the majority of the interventions were evaluated over a short time period. Secondly, a significant proportion of studies were conducted in patients with NERD, and the diagnosis of GERD relied solely on patients’ reported symptoms in most studies. As the typical symptoms of GERD can also manifest in non-GERD conditions, this diagnosis approach may be suboptimal. Thirdly, obesity was found to be common, which could potentially confound study outcomes due to its established association with GERD development, and as it is a main reason for prescribing dietary modifications. [48,49,50] Additionally, the majority of both the RCT and non-RCT studies were categorized as low quality. In clinical practice, GERD patients may respond differently even to the same dietary intervention. Hence, a personalized approach may be required to achieve the goals of these dietary interventions for each patient. Unnecessary dietary restrictions should be avoided, as the effect of most dietary interventions could not be confirmed by current evidence and may result in a reduced QoL and inadequate nutritional intake, especially in malnourished patients.

## 5. Conclusions

A meta-analysis of low-carbohydrate diets and eating speed interventions was performed in the present study. While the former showed significant improvement in esophageal acid exposure, a slow eating speed did not result in a significant difference in reflux events compared to a fast eating speed. Intervention studies to confirm the benefits of the other dietary interventions are lacking. Moreover, several limitations were identified in the studies, and it is therefore challenging to draw a firm conclusion. An individualized approach to dietary counseling is still needed for patients. High-quality, long-term RCTs are still required to confirm the effects of dietary interventions in GERD patients.

## Figures and Tables

**Table 1:** Characteristics of the 16 dietary interventions included in the 21 studies.

Study	Study Type	Country	Sample Size	Population	Age (Years)	Gender (F/M, % F)	BMI (kg/m^2^)	Intervention	Duration of Intervention	Quality Score
**Low-carbohydrate diets**										
Austin et al., 2006 [14]	Single-arm intervention study	USA	8	GERD (symptoms) and obesity (BMI > 30 kg/m^2^)	Mean (SD), 40 (10)	8/0, 100%	Mean (SD), 43.5 (9.2)	Pre- and post- low-carbohydrate diet (<20 g/day)	3–6 days	1
Wu et al., 2018 [15]	Non-randomized, cross-over	Taiwan	12	GERD (symptoms)	Mean (SD), 43.5 (9.2)	5/7, 71.4%	Mean (SD), 24.3 (3.8)	Low- vs. high-carbohydrate diets (84.8 g vs. 178.8 g)	One meal (6 h wash out period)	2
Gu et al., 2022 [16]	RCT	USA	98	GERD (symptoms)	Mean (SD), 60 (12.5)	16/79, 16.8%	Mean (SD), 32.7 h (5.4)	High total/high simple carbohydrate (HTHS), high total/low simple carbohydrate (HTLS), low total/high simple carbohydrate (LTHS), or low total/low simple carbohydrate (LTLS) diets	9 weeks	4
**High-fat diets**										
Penagini et al., 1998 [17]	RCT, cross-over	Italy	14	GERD (endoscopy and/or pH monitoring) 6 RE 8 abnormal pH monitoring	Range, 23–60	4/10, 28.6%	N/A	High-fat meal vs. balanced meal	One meal (2-day wash out period)	2
Fan et al., 2018 [12]	RCT, cross-over	China	27	GERD (symptoms) 15 RE 12 NERD	RE: mean (SD), 50.9 (7.5) NERD: mean (SD), 46.8 (11.3)	RE 9/6, 60% NERD 5/7, 41.7%	RE: mean (SD), 24.4 (2.2) NERD: mean (SD), 23.3 (1.5)	High-fat meal vs. standard meal	One meal (5 h and 17.5 h wash out period)	3
**Low-FODMAP diets**										
Rivière et al., 2021 [18]	RCT	France	31	GERD (symptoms) and PPI refractory	Median (range), 45 (39–51)	17:14, 55%	N/A	Low-FODMAP diet vs. usual dietary advice	4 weeks	3
Plaidum et al., 2022 [8]	RCT, cross-over	Thailand	8	GERD (symptoms) and overlapping IBS (non-constipation)	Mean (SD), 57 (13)	6:2, 75%	Mean (SD), 23.3 (2.7)	Rice noodle vs. wheat noodle meals for breakfast and lunch	Two meals (1-week wash out period)	3
**Eating speed**										
Bor et al., 2013 [19]	RCT, cross-over	Turkey	46	GERD (symptoms)	Median, 43	32/14, 69.6%	N/A	5 min vs. 30 min	One meal (1-day wash out period)	2
Valitova et al., 2013 [20]	RCT, cross-over	Turkey	60	GERD (symptoms)	Mean (SD), 43.5 (10.8)	39/21, 65%	N/A	5–10 min vs. 25–30 min	One meal (1-day wash out period)	2
Bor et al., 2017 [21]	RCT, cross-over	Turkey	26	GERD (symptoms) and obesity (BMI > 30 kg/m^2^)	Mean (SD), 46 (12)	26/0, 100%	Mean (SD), 39.9 (8.4)	5 min vs. 30 min	One meal (1-day wash out period)	2
**Other dietary interventions**										
Bove et al., 2003 [22]	RCT, cross-over	Sweden	9	GERD (symptoms and pH monitoring)	Median (range), 40 (25–54)	4/5, 44.4%	N/A	High-nitrate vs. nitrate-free diets	4 days (2-week wash out period)	4
Pereira et al., 2006 [23]	RCT	Brazil	351	GERD (symptoms)	Mean (range), 44 (18–88)	210/141, 59.8%	N/A	Dietary supplementation containing melatonin, l-tryptophan, vitamin B6, folic acid, vitamin B12, methionine, and betaine vs. 20 mg omeprazole	40 days	3
Lim et al., 2011 [24]	Single-arm intervention study	Singapore	25	GERD (symptoms and endoscopy) NERD	Mean (SD), 44.8 (2.4)	2/23, 8%	N/A	Pre- and post- curry ingestion	One meal	6
Panahi et al., 2015 [25]	RCT	Iran	79	GERD (symptoms)	Mean (SD), 47 (17)	45/34, 57%	Mean (SD), 25.4 (4.5)	Aloe vera vs. omeprazole vs. ranitidine	4 weeks	3
Fan et al., 2018 [12]	RCT, cross-over	China	27	GERD (symptoms) 15 RE 12 NERD	RE: mean (SD), 50.9 (7.5) NERD: mean (SD), 46.8 (11.3)	RE 9/6, 60% NERD 5/7, 41.7%	RE: mean (SD), 24.4 (2.2) NERD: mean (SD), 23.3 (1.5)	Functional food vs. standard meal	One meal (5 h and 17.5 h wash out period)	3
Morozov et al., 2018 [26]	Single-arm intervention study	Russia	30	GERD (symptoms and endoscopy) NERD with low dietary fiber intake	Mean (SD), 34.7 (9.3)	12/18, 40%	Mean (SD), 26.7 (6.9)	Pre- and post- psyllium 15 g per day	10 days	3
Fatani et al., 2020 [27]	RCT	USA	51	GERD (symptoms)	Intervention: median (range), 30 (18–55) Control: median (range), 24 (19–56)	37/14, 72.5%	N/A	Fermented soy vs. placebo	3 weeks	5
Beckett et al., 2020 [28]	RCT	Australia	40	GERD (symptoms)	Mean (SD), 46.0 (12.6)	26/14 (65%)	Mean (SD), 32.6 (8.7)	Sugar cane flour vs. placebo	3 weeks	5
Triadafilopoulos et al., 2020 [29]	Single-arm intervention study	USA	66	GERD (symptoms) 34 normal AET 32 abnormal AET	Median (range), 51 (20–87)	36/30, 54%	Normal AET: mean (SE), 24.7 (1.9) Abnormal AET: mean (SE), 26 (1.1)	Pre- and post- restricted (anti-reflux) diet	2 days	3
Yousaf et al., 2021 [30]	Single-arm intervention study	USA	20	GERD (symptoms) and PPI refractory	Mean (SD), 49.95 (12.74)	16/4, 80%	Mean (SD), 35.24 (9.04)	Pre- and post- low-nickel diet	8 weeks	2
Mendoza-Martínez et al., 2022 [31]	RCT	Mexico	95	GERD (symptoms)	non-caloric sweeteners (NCS): mean (SD), 22 (3.1) Non-caloric sweetener-free diet (NCS-f): mean (SD), 22 (3.2)	58/37, 61%	NCS: mean (SD), 23.9 (3.1) NCS-f: mean (SD), 24.16 (3.8)	NCS vs. NCS-f	5 weeks	3
Polese et al., 2022 [32]	RCT, cross-over	Italy	40	GERD (symptoms) and 50% of time following coffee consumption	Mean (SD), 41.5 (12)	16/24, 40%	Mean (SD), 25.5 (4)	Standard coffee vs. dewaxed coffee	2 weeks (2-week wash out period)	3

Abbreviation: F, female; M, male; GERD, gastroesophageal reflux disease; BMI, body mass index; SD, standard deviation; RCT, randomized controlled trial; RE, reflux esophagitis; N/A, not assessed; NERD, nonerosive reflux disease; PPI, proton pump inhibitor; FODMAP, fermentable oligosaccharides, disaccharides, monosaccharides, and polyols; IBS, irritable bowel syndrome; AET, esophageal acid exposure time; SE, standard error.

**Table 2:** Summary of the results of the dietary interventions in GERD patients.

Study	Intervention	Control	Outcomes		
			GERD Symptoms	pH Monitoring Measurement	Quality of Life
**Low-carbohydrate diets**					
Austin et al., 2006 [14]	Pre- and post-low-carbohydrate diet (daily carbohydrate intake < 20 g/day) for 3–6 days	N/A	- Significant decrease in the GERD Symptom Assessment Scale–Distress Subscale (GSAS-ds) score after low-carbohydrate diet (mean (SE), 1.28 (0.15) vs. 0.72 (0.12); *p* = 0.0004)	- Significant decrease in 24 h esophageal acid exposure time (AET) after low-carbohydrate diet (mean (SE), 5.1% (1.3) vs. 2.5% (0.6); *p* = 0.022) - Significant decrease in Johnson–DeMeester Score after a low-carbohydrate diet. (mean (SE), 34.7 (10.1) vs. 14.0 (3.7); *p* = 0.023)	N/A
Wu et al., 2018 [15]	Low-carbohydrate diet, 500 mL liquid meal (474.4 kcal, 10.4 g protein, 10.4 g fat, 84.8 g carbohydrate)	High-carbohydrate diet, 500 mL liquid meal (850.4 kcal,10.4 g protein, 10.4 g fat, 178.8 g carbohydrate)	- Higher heartburn and acid regurgitation post high-carbohydrate diet	- Higher Johnson–DeMeester scores post high-carbohydrate diet (mean (SD), 39.7 (11.0) vs. 14.3 (5.3); *p* = 0.019) - Higher numbers of reflux periods post high-carbohydrate diet (mean (SD), 12.7 (2.1) vs. 7.1 (2.3); *p* = 0.026)	N/A
Gu et al., 2022 [16]	High total/low simple carbohydrate (HTLS), low total/high simple carbohydrate (LTHS), and low total/low simple carbohydrate (LTLS) diets for 9 weeks	High total/high simple carbohydrate diet (HTHS) for 9 weeks	- Significant reduction in total GERD-Q score between pre- and post intervention within HTLS (mean (SD), −3.1 (3.6)), LTHS (−3.7 (3.4)) and LTLS (−3.5 (3.9)), non-significant reduction in HTHS (−1.4 (1.1))	- Significant reduction in AET between pre- and post intervention within HTLS (median (interquartile range (IQR), −3.0% (1.3 to −6.2)) and LTHS (−2.7% (0.5 to −6.6)), non-significance in HTHS (0.5% (−1.0 to 3.7) and LTLS (0.6% (−1.0 to 3.5). - Significant reduction in total reflux episodes between pre- and post intervention within HTLS (median (IQR), −14.8 (−56.8 to 12.0)) and LTHS (−12.7 (−64.2 to 14.0)), non-significance in HTHS (18.7 (−30.0 to 77.5)) and LTLS (6.0 (−14.4 to 31.6)).	N/A
**High-fat diets**					
Penagini et al., 1998 [17]	High-fat meal (44 g fat) Carbohydrate (C): Fat (F): Protein (P), 39:52:9% 760 kcal, 450 mL (150 mL Ensure, 150 mL lipofundin, 150 mL saline) Infused 40 mL/min into stomach Plus eating 1 sandwich and 150 mL Ensure Position: 7 recumbent and 7 sitting	Balanced meal (20 g fat) C:F:P 60:24:16% 755 kcal, 450 mL (450 mL Ensure) Infused 40 mL/min into stomach Plus eating 1 sandwich and 150 mL Ensure Position: 7 recumbent and 7 sitting	N/A	- No significant difference in transient lower esophageal sphincter relaxations (TLESR) between groups - No significant difference in Basal lower esophageal pressure between groups - No significant difference in AET at 3 h between groups: recumbent (mean (SE): 16.5% (7.5) vs. 19.5% (6.5) sitting (mean (SE): 6.3% (2.4) vs. 8.6% (2.9) - No significant difference in rate of reflux episodes per hour recumbent (mean (SE): 5.2 (1.9) vs. 4.8 (1.7) sitting (mean (SE): 2.4 (0.7) vs. 3.9 (1.1)	N/A
Fan et al., 2018 [12]	High-fat meal (53.7 g fat) C:F:P, 29.1:60.6:9.3% 800 kcal, 800 mL Position: upright	Standard meal (22.2 g fat) C:F:P 12.3:25:62.6% 800 kcal, 800 mL Position: upright	- No significant difference in number of postprandial reflux symptoms RE group (median (IQR): 1 (0–1) vs. 1 (0–2) NERD group (median (IQR): 1 (0–2) vs. 3 (1–4)	- Significant difference in AET at 4 h RE group: (median (IQR): 5.2% (0.5–22.4) vs. 4.0% (0–10.5) - No significant difference in percentage of time pH < 4 in gastric fundus	N/A
**Low-FODMAP diets**					
Rivière et al., 2021 [18]	Low-FODMAP diet for 4 weeks	Usual dietary advice for 4 weeks	- No significant difference in reduction in a Reflux Disease Questionnaire (RDQ) score ≤ 3 between two groups (37.5% vs. 20%; *p* = 0.43)	- No significant difference in total acid exposure between two groups (median (IQR), 0.9 (0.0–1.9) vs. 1.0 (0.2–2.0); *p* = 0.88) - No significant difference in total reflux events between two groups (median (IQR), 46 (35–61) vs. 51 (28–99); *p* = 0.41)	N/A
Plaidum et al., 2022 [8]	Rice noodle meal (low FODMAPs) for breakfast and lunch	Wheat noodle meal (high FODMAPs) for breakfast and lunch	- Significantly higher regurgitation severity scores after wheat meal compared to rice meal (median (IQR), 1.5 (0.0–6.1) vs. 0.3 (0.0–0.9); *p* < 0.05)	- Significantly higher number of TLESR events in the 2 h after wheat meal compared to rice meal (mean (SD), 5.00 (0.68) vs. 1.88 (0.30); *p* = 0.01)	N/A
**Eating speed**					
Bor et al., 2013 [19]	5 min Standard meal (a double cheeseburger, 1 banana, 100 g yogurt, and 200 mL water) 744 kcal C:F:P = 37.6:41.2:21.2%	30 min Standard meal (a double cheeseburger, 1 banana, 100 g yogurt, and 200 mL water) 744 kcal C:F:P = 37.6:41.2:21.2%	N/A	- No significant difference in total reflux events in 3 h (number, 753 vs. 733) - No significant difference of reflux events in the first, second, and third hour	N/A
Valitova et al., 2013 [20]	5–10 min (mean (SD) 8.4 (2.4) minutes) Balanced meal (a double cheeseburger, 1 banana, 100 g yogurt, and 200 mL water) C:F:P = 37.6:41.2:21.2%	25–30 min (mean (SD) 27.7 (4) minutes) Balanced meal (a double cheeseburger, 1 banana, 100 g yogurt, and 200 mL water) C:F:P = 37.6:41.2:21.2%	- No significant difference in reflux symptoms in 3 h (heartburn or regurgitation) All patients: number 100 vs. 113 Pathologic pH monitoring patients: number 48 vs. 54	N/A	N/A
Bor et al., 2017 [21]	5 min Standard meal (a double cheeseburger, 1 banana, 100 g yogurt, and 200 mL water) 744 kcal C:F:P = 37.6:41.2:21.2%	30 min Standard meal (a double cheeseburger, 1 banana, 100 g yogurt, and 200 mL water) 744 kcal C:F:P = 37.6:41.2:21.2%	N/A	- No significant difference in total reflux events in 3 h All patients: number (mean), 715 (11.9) vs. 668 (11.1) Pathologic pH monitoring patients: number (mean), 418 (19.0) vs. 418 (19.0) - No significant difference in total reflux time in 3 h All patients: minutes (mean), 1007 (16.8) vs. 866 (14.4) Pathologic pH monitoring patients: minutes (mean), 716 (32.5) vs. 627 (28.5)	N/A
**Other dietary interventions**					
Bove et al., 2003 [22]	Nitrate capsule for lunch and dinner (200 mg, 50% recommended dose and 3–4 times the mean nitrate in Swedish diets) Plus nitrate/nitrite-free diet for 4 days	Placebo capsule for lunch and dinner Plus nitrate/nitrite-free diet for 4 days	N/A	- No significant difference in TLESR time (second) between groups Supine: mean (SD), 92.1 (78.3) vs. 93.9 (46.1) Sitting after gastric distension: mean (SD), 183.9 (79.9) vs. 103.9 (85.7) - No significant difference in number of TLESR in 30 min Supine: mean (SD), 4.9 (4.3) vs. 4.9 (2.5) Sitting after gastric distension: mean (SD), 8.0 (3.1) vs. 6.6 (6.2) - No significant difference in AET (mean (SD), 6.0% (4.1) vs. 7.4% (7.4) - No significant difference in number of reflux episodes (mean (SD), 39 (22.5) vs. 35 (17.5)	N/A
Pereira et al., 2006 [23]	Dietary supplement (melatonin (6 mg), tryptophan (200 mg), vitamin B12 (50 lg), methionine (100 mg), vitamin B6 (25 mg), betaine (100 mg), and folic acid (10 mg)) for 40 days	20 mg omeprazole for 40 days	- Significant reduction in symptoms in the dietary supplement group (100% vs. 65.7%; *p* = 0.001)	N/A	N/A
Lim et al., 2011 [24]	400 mL (15 patients) or 800 mL (10 patients) of cooked curry suspension ingested over 5 min	N/A	- Significant higher total symptom score of 6 GERD symptoms (each analog scale 0–10) after curry ingestion at 15, 30, 60, 90, 120, 150 min (*p* < 0.005), non-significance at 180 min - Significant higher total symptom score after 800 mL curry ingestion compared to 400 mL (mean (SD) 14.1 (3.7) vs. 3.3 (1.0) at 180 min; *p* < 0.05	- Significant higher AET at 3 h after curry ingestion Pre- vs. post- 400 mL: mean (SD), 5.4 (1.2) vs. 11.8 (2.2); *p* = 0.007 Pre- vs. post- 800 mL: mean (SD), 6.4 (3.3) vs. 20.5 (6.8); *p* = 0.007 Pre- vs. post- overall: mean (SD), 5.8 (1.4) vs. 15.3 (3.1); *p* < 0.001	N/A
Panahi et al., 2015 [25]	Aloe vera syrup (10 mL once a day) for 4 weeks	Omeprazole (20 mg once a day) or ranitidine tablet (150 mg twice a day) for 4 weeks	- Significant reduction in the frequency of heartburn and regurgitation in all groups but less reduction in heartburn in the aloe vera group	N/A	N/A
Fan et al., 2018 [12]	Functional food (22.4 g fat) C:F:P14.6:25:60.4% 800 kcal, 800 mL Position: upright	Standard meal (22.2 g fat) C:F:P 12.3:25:62.6% 800 kcal, 800 mL Position: upright	- Significantly lower number of postprandial reflux symptoms after functional food in NERD group (median (IQR), 0 (0–1) vs. 3 (1–4)), but non-significance in RE group (median (IQR), 1 (0–2) vs. 1 (0–2))	- No significant difference in AET at 4 h between 2 groups (median (IQR), 4.3 (0–26.5) vs. 4.0 (0–10.5)) - No significant difference in percentage of time pH < 4 in gastric fundus between 2 groups	N/A
Morozov et al., 2018 [26]	Psyllium 15 g per day for 10 days	N/A	- Significant improvement of heartburn in 60% of patients and decreased GERD-Q scores after psyllium ingestion (mean (SD)), 10.9 (1.7) vs. 6.0 (2.3))	- Significant reduction in the number of reflux episodes after psyllium ingestion (mean (SD), 67.9 (17.7) vs. 42.4 (13.5); *p* < 0.001) without change of 24 h pH below 4	N/A
Fatani et al., 2020 [27]	Fermented soy (1 sachet) after heartburn symptoms, can repeat with second dose if heartburn persists after 30 min, and can repeat with third dose or OTC medication if heartburn persists after 30 min, for 3 weeks	Maltodextrin (1 sachet) after heartburn symptoms, can with repeat second dose if heartburn persists after 30 min, and can repeat with third dose or OTC medication if heartburn persists after 30 min, for 3 weeks	- No significant difference in heartburn severity after ingestion at 5, 15, 30 min, evaluated by Likert-like scale - No significant difference in heart burn frequency (number per week) between baseline and intervention	N/A	- Significantly better GERD quality of life score in some items compared between intervention and baseline: “I found it inconvenient to have to take medications regularly because of acid reflux and heartburn symptoms” (mean (SD), − 1.0 (1.3) vs. − 0.04 (1.8); *p* < 0.05) “I was afraid to eat too much because of acid reflux and heartburn symptoms” (mean (SD), − 1.4 (1.3) vs. −0.2 (1.7); *p* < 0.05) “I was unable to concentrate on my work because of acid reflux and heartburn symptoms” (mean (SD), − 0.9 (1.6) vs. −0.3 (1.0); *p* < 0.05) “Acid reflux and heartburn symptoms disturbed my after-meal activities or rest” (mean (SD), − 1.6 (1.5) vs. −0.7 (1.5); *p* < 0.05) - No significant difference in overall score
Beckett et al., 2020 [28]	3 g/day of prebiotic whole-plant sugarcane flour (PSCF) after morning and evening meal for 3 weeks	3 g/day of cellulose after morning and evening meal for 3 weeks	- Significantly higher number of patients with improved heartburn symptoms in PSCF group (13 (65%) vs. 5 (25%); *p* = 0.039) - Significantly higher number of patients with improved regurgitation symptoms in PSCF group (11 (55%) vs. 1 (5%); *p* = 0.001) - Significantly higher number of patients with improved total Gastroesophageal Reflux Disease-Health Related Quality of Life (GERD-HRQL) symptom scores in PSCF group (13 (65%) vs. 4 (20%); *p* = 0.015) - Significantly higher GERD-HRQL after PSCF ingestion Heartburn score: −2.2; 95% CI −4.2 to −0.14; *p* = 0.037 Total symptom score: −3.7; 95% CI −7.2 to −0.11; *p* = 0.044	N/A	N/A
Triadafilopoulos et al., 2020 [29]	Restricted (anti-GER) diet through provided instructions and diet recommendations	N/A	- No significant difference in symptoms	- Significant reduction in AET at 48 h after the restricted diet in patients with abnormal AET (median, 10.5% (95% CI 8.9–12.6) vs. 4.5% (95% CI 3.1–7.3); *p* = 0.001), but non-significance in patients with normal AET (median, 3.2% (95% CI 1.9–4.0) vs. 2.6% (95% CI 0.8–3.4))	N/A
Yousaf et al., 2021 [30]	low-nickel diet for 8 weeks	N/A	- Significant decrease in total GERD-HRQL, heartburn, and regurgitation scores	N/A	N/A
Mendoza-Martinez et al., 2022 [31]	Non-caloric sweetener-free (NCS-f) diet with less than 10 mg/day non-caloric sweetener (NCS)	NCS diet with 50–100 mg/day NCS (80% sucralose and 20% aspartame, acesulfame K, and saccharin)	- Significant improvement of burning and retrosternal pain in the NCS-f group (15% of participants in pre-treatment to 0% of post-treatment; *p* = 0.02)	N/A	N/A
Polese et al., 2022 [32]	Dewaxed coffee (DC) for 2 weeks	Standard coffee (SC) for 2 weeks	- Significant increase in both heartburn-free days and regurgitation-free days during DC compared to SC	N/A	- Significant improvement in quality of life in DC compared to SC

Abbreviation: GERD, gastroesophageal reflux disease; N/A, not assessed; SE, standard error; SD, standard deviation; RE, reflux esophagitis; IQR, interquatile range; NERD, nonerosive reflux disease; FODMAP, fermentable oligosaccharides, disaccharides, monosaccharides, and polyols; TLESR, transient lower esophageal sphincter relaxation; OTC, over-the-counter; CI, confidence interval.
